# Efficacy of Sodium-Glucose Cotransporter 2 (SGLT2) Inhibitors in Heart Failure With Preserved Ejection Fraction: A Systematic Review

**DOI:** 10.7759/cureus.84129

**Published:** 2025-05-14

**Authors:** María Victoria Santos Guzmán, Daimi Rivera, Isabella Marian Reyna Guerrero, Alfida Luisa Fernandez Rodriguez, Rania Azcona, Diego Escobar Batista, Gabriela Montano Diaz, Luis Carlos García Zuluaga, Victor Santos Rosario

**Affiliations:** 1 Medicine, Universidad Iberoamericana (UNIBE), Santo Domingo, DOM; 2 Faculty of Medicine, Universidad Iberoamericana (UNIBE), Santo Domingo, DOM; 3 Internal Medicine, Hospital General de la Plaza de la Salud (HGPS), Santo Domingo, DOM

**Keywords:** cardiovascular mortality, dapagliflozin, heart failure hospitalization, heart failure with preserved ejection fraction, quality of life, randomized controlled trial, sodium–glucose transporter 2 inhibitors

## Abstract

Heart failure with preserved ejection fraction (HFpEF) represents a growing clinical challenge with limited therapeutic options. Sodium-glucose cotransporter 2 (SGLT2) inhibitors have recently emerged as a promising intervention; however, their impact across diverse HFpEF populations requires further clarification. We conducted a systematic review of four completed randomized controlled trials (RCTs) - CANDLE (Canagliflozin Anti-inflammatory and Metabolic Effects), EMPEROR-Preserved (Empagliflozin Outcome Trial in Patients with Chronic Heart Failure with Preserved Ejection Fraction), the JCI 2021 Empagliflozin HFpEF trial (a Japan Cardiovascular Initiative study assessing empagliflozin in heart failure with preserved ejection fraction), and a multi-agent SGLT2 inhibitor study (empagliflozin, dapagliflozin, and canagliflozin vs. standard of care) - plus one ongoing multicenter study, HELD-HF (Heart Failure with Preserved Ejection Fraction and Empagliflozin in a Long-term Diabetes Population), encompassing approximately 13,400 participants with HFpEF, to evaluate the efficacy and safety of SGLT2 inhibition.

In EMPEROR-Preserved (*n* = 5,792; median follow-up 26.2 months), empagliflozin reduced heart failure hospitalizations from 11.1% to 8.3% and cardiovascular mortality from 16.2% to 13.4% (hazard ratio (HR) 0.75; 95% confidence interval (CI) 0.68-0.84). In a multi-agent trial (*n* = 1,253; 18 months), SGLT2 inhibitors lowered cardiovascular mortality (4% vs. 5%) and HF hospitalizations (7% vs. 10%), with an absolute risk reduction of 1% and 3%, respectively, and improved patient-reported quality of life (mean Kansas City Cardiomyopathy Questionnaire (KCCQ) score increase of 8 points).

Functional capacity, assessed by the 6-minute walk test, improved by a mean of 25-30 m across completed studies, and KCCQ scores rose by 5-10 points. N-terminal pro-B-type natriuretic peptide (NT-proBNP) levels decreased by 10-20% (e.g., -10.4% vs. comparator in CANDLE). Safety profiles were favorable: serious adverse event rates were comparable to placebo (≈12% vs. 13%), genital infections occurred in ~2.5% vs. 0.5%, and symptomatic hypotension in 7% vs. 5% of participants. The ongoing HELD-HF trial (*n* = 112; 24 weeks) will further elucidate effects on left ventricular mass index and NT-proBNP in dialysis-dependent HFpEF patients. These findings support the integration of SGLT2 inhibitors into HFpEF management and highlight the need for further large-scale, long-term studies to optimize their therapeutic application.

## Introduction and background

Heart failure with preserved ejection fraction (HFpEF) accounts for nearly half of all heart failure cases in North America and Europe - estimates range from 45% to 55%, depending on cohort definitions, and is characterized by symptoms of heart failure despite a left ventricular ejection fraction (LVEF) ≥50%. Its prevalence is rising in parallel with the aging population and the increasing burden of comorbidities such as hypertension, obesity, and diabetes mellitus [1]. Patients with HFpEF experience substantial morbidity, including exercise intolerance and frequent hospitalizations, and carry a prognosis comparable to that of heart failure with reduced ejection fraction (HFrEF), with five-year mortality rates approaching 50% [2].

The pathophysiology of HFpEF is complex and heterogeneous, involving myocardial stiffening, impaired relaxation, and systemic endothelial dysfunction. Contributing mechanisms include oxidative stress, low-grade inflammation, and alterations in myocardial energetics, which collectively lead to diastolic dysfunction and elevated filling pressures [3]. Neurohormonal activation and comorbidity-driven processes further exacerbate ventricular-vascular uncoupling and impair cardiac reserve, ultimately limiting patients’ functional capacity [4].

Sodium-glucose cotransporter 2 (SGLT2) inhibitors, originally developed for glycemic control in type 2 diabetes, have demonstrated cardioprotective effects that extend beyond glucose lowering. Proposed mechanisms-natriuresis and osmotic diuresis, favorable renal hemodynamics, shifts in myocardial energy substrate utilization toward ketones, and attenuation of myocardial fibrosis and inflammation-are supported by preclinical and clinical data. Large randomized trials, such as EMPEROR-Preserved (Empagliflozin Outcome Trial in Patients With Chronic Heart Failure With Preserved Ejection Fraction) and DELIVER (Dapagliflozin Evaluation to Improve the LIVEs of Patients With Preserved Ejection Fraction Heart Failure), have reported significant reductions in the composite outcome of cardiovascular death and heart failure hospitalization in HFpEF populations treated with empagliflozin or dapagliflozin [5-6].

Despite these promising findings, current reviews have not fully addressed the heterogeneity of treatment effects across different SGLT2 agents, the underrepresentation of key subgroups (e.g., patients with advanced chronic kidney disease or atrial fibrillation), or the balance of clinical outcomes versus biomarker and patient-reported endpoints. Furthermore, only one ongoing trial (Heart Failure with Preserved Ejection Fraction and Empagliflozin in a Long-term Diabetes Population (HELD-HF)) is examining structural cardiac changes and biomarker responses in dialysis-dependent HFpEF patients [7]. Therefore, we conducted a systematic review of randomized controlled trials (RCTs) and ongoing studies to synthesize the available evidence regarding the efficacy and safety of SGLT2 inhibitors in HFpEF. Our review prioritizes clinical outcomes (primary focus) while also systematically evaluating biomarker changes and patient-reported measures as co-primary endpoints, and adheres to the Preferred Reporting Items for Systematic Reviews and Meta-Analyses (PRISMA) 2020 guidelines. These findings aim to inform clinical practice and guide future research directions in the management of HFpEF.

## Review

Methodology

We conducted a systematic review following the PRISMA 2020 guidelines. A comprehensive search of the PubMed database was performed from inception through April 24, 2025. The search strategy combined Medical Subject Headings (MeSH) and free-text terms related to HFpEF and SGLT2 inhibitors. Filters were applied to restrict results to studies classified as "Randomized Controlled Trial" and "Clinical Trial."

Two independent reviewers (MVS and DR) screened titles and abstracts for relevance. Full-text articles meeting the eligibility criteria were subsequently retrieved for detailed evaluation. Any discrepancies between reviewers were resolved through discussion. If consensus could not be reached, a third reviewer (VSR) adjudicated the decision.

PICO framework

The PICO framework is summarized in Table 1.

**Table 1:** PICO framework. LVEP, left ventricular ejection fraction; HFpEF, heart failure with preserved ejection fraction; SGLT2, sodium-glucose cotransporter 2; NT-proBNP, N-terminal pro–B-type natriuretic peptide

Elements	Description
P (Population)	Adults (≥18 years) diagnosed with heart failure with preserved ejection fraction (LVEF ≥50%)
I (Intervention)	Administration of SGLT2 inhibitors (e.g., dapagliflozin, empagliflozin, canagliflozin, ertugliflozin)
C (Comparison)	Placebo or standard-of-care treatment
O (Outcomes)	Primary outcomes: cardiovascular mortality, heart failure hospitalizations; Secondary outcomes: improvement in quality of life (Kansas City Cardiomyopathy Questionnaire score), functional capacity (six-minute walk test), and biomarker levels (NT-proBNP)

Inclusion Criteria

We included English-language RCTs and clinical trials published within the last 10 years that enrolled adult patients (≥18 years) diagnosed with HFpEF (LVEF ≥50%, as defined by echocardiography or other imaging modalities). Eligible studies had to evaluate at least one SGLT2 inhibitor compared to a placebo or standard-of-care. Studies were required to report at least one relevant clinical endpoint, such as cardiovascular mortality, heart failure hospitalization rates, patient-reported quality of life (e.g., KCCQ), functional capacity, or biomarker changes.

Exclusion Criteria

We excluded studies that primarily enrolled populations with HFrEF; those with non-randomized or observational designs; animal or in vitro experiments; pharmacokinetic/pharmacodynamic studies without clinical outcomes; case reports or case series with fewer than five patients (unless providing exceptionally detailed mechanistic insights); conference abstracts or poster presentations without full-text availability; and trials that did not report predefined cardiovascular or patient-centered outcomes.

Risk-of-bias assessment (Cochrane Risk of Bias tool)

The methodological quality and risk of bias of each included RCT were independently assessed by two reviewers (MVS, DR) using the Cochrane Risk of Bias 2.0 tool [6]. This tool evaluates six domains: (1) Random Sequence Generation, (2) Allocation Concealment, (3) Blinding of Participants & Personnel, (4) Blinding of Outcome Assessment, (5) Incomplete Outcome Data, and (6) Selective Reporting. Each domain is rated as *low risk*, *some concerns*, or *high risk*. An overall risk-of-bias assessment is then assigned to each study, with any disagreements resolved through discussion or, if necessary, adjudication by a third reviewer (VSR). The detailed risk-of-bias assessment for each study is summarized in Table 2.

**Table 2:** Cochrane risk-of-bias assessment.

Study	Random Sequence Generation	Allocation Concealment	Blinding of Participants and Personnel	Blinding of Outcome Assessment	Incomplete Outcome Data	Selective Reporting
Tanaka et al. (2020) [7]	Low risk	Low risk	Low risk	Low risk	Low risk	Low risk
Böhm et al. (2022) [8]	Low risk	Low risk	Low risk	Low risk	Low risk	Low risk
Echouffo-Tcheugui et al. (2021) [9]	Low risk	Low risk	Low risk	Low risk	Low risk	Low risk
Greene et al. (2024) [10]	Low risk	Low risk	Low to moderate risk	Low risk	Low risk	Low risk
Yan et al. (2024) [11]	Low risk	Low risk	Low risk	Low risk	Low risk	Low risk

Interpretation of risk-of-bias assessment

The risk of bias was evaluated using the Cochrane Risk of Bias tool. Overall, study quality was high, with most domains judged at low risk; only Greene et al. [10] raised some concerns due to partial unblinding in outcome assessment. We briefly described the evidence underpinning each domain rating and summarized the global risk for each study.

Random Sequence Generation

Evidence: Tanaka et al. [7] and Yan et al. [11] both reported the use of a computer-generated random number table. Böhm et al. [8] described central randomization via a secure web-based system; Echouffo-Tcheugui et al. [9] used block randomization with concealed block sizes; Greene et al. utilized a computer algorithm with allocation codes held off-site [10].

Overall, all studies had a low risk for sequence generation, as methods were clearly described and robustly implemented [7-11].

Allocation Concealment

Evidence: Each trial stated that envelope-based or web-based systems were used, with opaque, sequentially numbered envelopes [7-9] or centralized allocation [10,11], ensuring that enrolling investigators could not predict assignments.

Overall, there was a low risk across all studies.

Blinding of Participants and Personnel (Performance Bias)

Evidence: All five studies employed placebo controls identical in appearance and taste, with treatment codes maintained by an independent pharmacy team. Trial protocols explicitly note that both participants and administering staff were unaware of group assignments.

Overall, there was a low risk across all studies.

Blinding of Outcome Assessment (Detection Bias)

Evidence: In studies by Tanaka et al. [7], Böhm et al. [8], Echouffo-Tcheugui et al. [9], and Yan et al. [11], laboratory analysts and data adjudicators were blinded to treatment allocation. Greene et al. [10] reported that one of two adjudicators became unblinded during a quality-control review, introducing a low-to-moderate risk of detection bias.

Overall, four studies were at low risk, while Greene et al. [10] were judged to have *some concerns*.

Incomplete Outcome Data (Attrition Bias)

Evidence: Attrition rates were below 5% in all trials, with reasons for withdrawal fully documented and balanced between groups. Intention-to-treat analyses were performed in each case.

Overall, all five studies were at low risk.

Selective Reporting (Reporting Bias)

Evidence: Protocols were available for all trials, and prespecified primary and secondary endpoints were reported in full in the final publications. No deviations from the analysis plan were noted.

Overall, each study had a low risk.

Global Risk Summary

Low risk of bias: Tanaka et al. [7], Böhm et al. [8], Echouffo-Tcheugui et al. [9], Yan et al. [11]

Some concerns: Greene et al. [10], driven by a moderate risk in blinding of outcome assessment

Study selection and data extraction

Two reviewers (MVS, DR) independently screened titles and abstracts using Rayyan. Full-text articles meeting the eligibility criteria were subsequently reviewed. Discrepancies in study selection were resolved through discussion or adjudication by a third reviewer (VSR). Extracted data fields included: study design, sample size, patient demographics, heart failure diagnostic criteria, intervention details (type and dosage of SGLT2 inhibitor), comparator characteristics, duration of follow-up, primary and secondary outcomes, and study quality assessment using the Cochrane Risk of Bias tool.

MeSH terms

("Heart Failure, Diastolic"[MeSH] OR "heart failure with preserved ejection fraction"[Title/Abstract] OR "HFpEF"[Title/Abstract]) AND ("Sodium-Glucose Transporter 2 Inhibitors"[MeSH] OR "SGLT2 inhibitors"[Title/Abstract] OR "dapagliflozin"[Title/Abstract]) AND ("Randomized Controlled Trial"[Publication Type] OR "Clinical Trial"[Publication Type])

Data availability

The data supporting the findings of this study are available from the corresponding author upon reasonable request.

Data synthesis

Given the clinical and methodological heterogeneity across the included RCTs of SGLT2 inhibitors in HFpEF, data were synthesized narratively. This synthesis was organized by type of intervention (e.g., dapagliflozin vs. placebo) and outcome category (primary vs. secondary). A meta-analysis was not performed due to the variability in study designs, outcome measures, and statistical methodologies across studies.

Reporting bias and certainty of evidence

Due to the limited number of included studies, a formal assessment of reporting bias, such as funnel plot analysis, was not conducted. The certainty of the evidence for each outcome was qualitatively assessed, considering factors such as risk of bias, consistency of findings, directness of evidence, and precision of effect estimates. These factors were integrated to provide an overall appraisal of the strength of the evidence supporting each outcome.

Results

**Figure 1:**
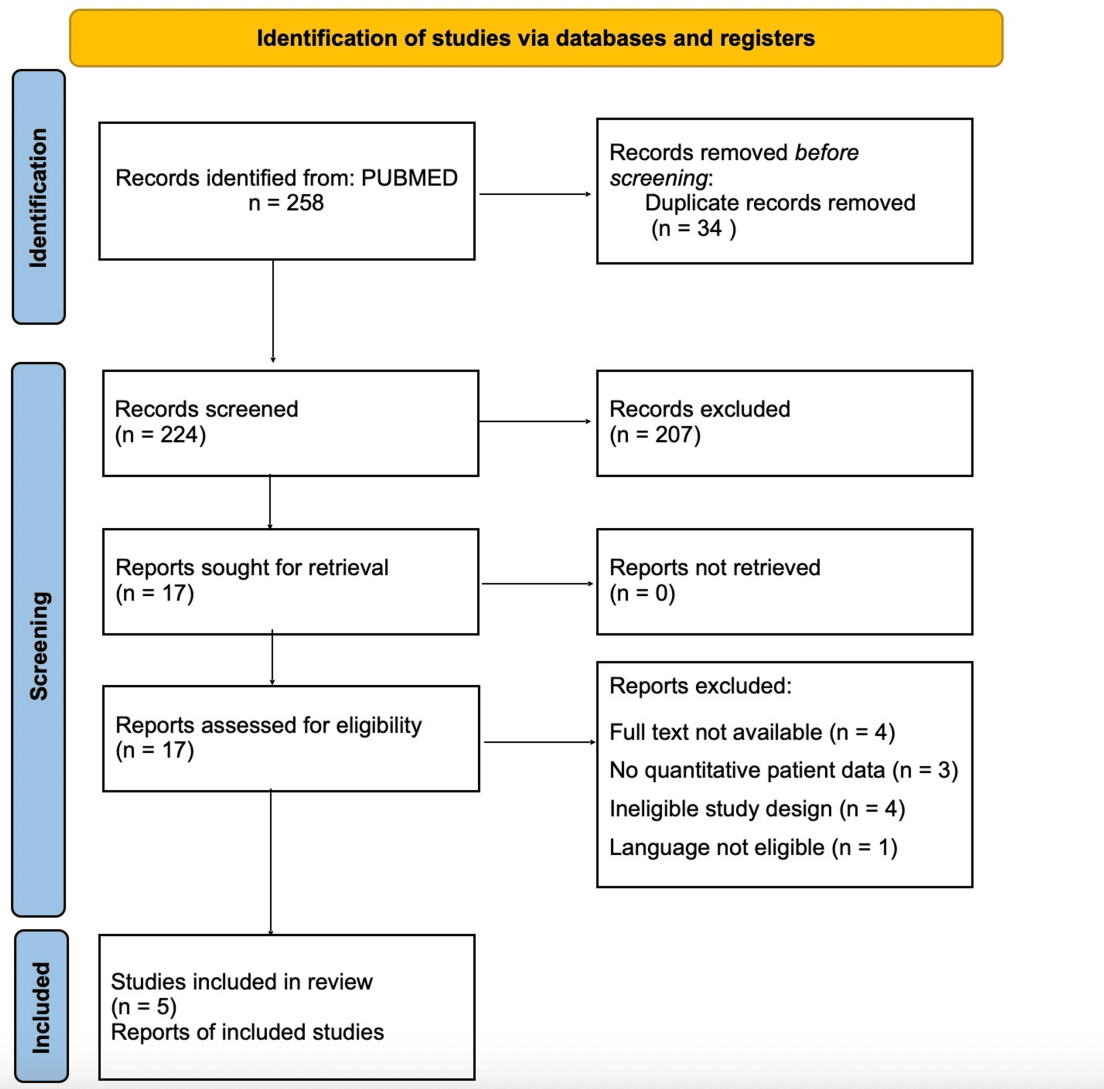
PRISMA 2020 flowchart. PRISMA, Preferred Reporting Items for Systematic Reviews and Meta-Analyses

A total of 258 articles were retrieved from PubMed using the predefined search strategy. After removing 34 duplicates, 224 articles were screened based on titles and abstracts. Seventeen articles were deemed potentially relevant, and their full texts were evaluated. Twelve studies were subsequently excluded for not meeting the inclusion criteria, leaving five high-quality studies for analysis. A complete PRISMA flow diagram summarizing the study selection process is provided in Figure 1.

The five included studies evaluated the efficacy of SGLT2 inhibitors in patients with HFpEF, as summarized in Table 3.

**Table 3:** Summary of clinical trials on SGLT2 inhibitors in heart failure: interventions, demographics, and key findings. LVEF, left ventricular ejection fraction; NYHA, New York Heart Association; IQR, interquartile range; HFpEF, heart failure with preserved ejection fraction; NT-proBNP, N-terminal pro-B-type natriuretic peptide

Study	Sample size	Intervention(s)	Comparator	Follow-up duration	Primary outcome(s)	Mean age (years)	Female (%)	Male (%)	Baseline LVEF	NYHA functional class distribution	Key findings
Tanaka et al. (2020) [7]	245	Canagliflozin 100 mg once daily for 24 weeks	Glimepiride (starting at 0.5 mg, titrated up to 6.0 mg as needed for 24 weeks)	24 weeks	Cardiovascular mortality and heart failure hospitalization; NT-proBNP changes	68.6 ± 10.1	25.3%	74.7%	Mean LVEF: 57.6 ± 14.6%	Class I: 64%; Class II: 34%; Class III: 2%; Class IV: 0%	Canagliflozin showed significant improvement in cardiovascular outcomes and NT-proBNP levels.
Böhm et al. (2022) [8]	5,792	Empagliflozin 10 mg daily	Placebo	26.2 months (median)	Reduction in cardiovascular mortality and heart failure hospitalizations; improvements in functional capacity and quality of life	72 (mean)	38%	62%	Approximately 57%	Predominantly NYHA Classes II and III	Empagliflozin significantly reduced cardiovascular mortality and heart failure hospitalizations, with notable improvements in functional capacity and quality of life.
Echouffo-Tcheugui et al. (2021) [9]	5,988	Empagliflozin 10 mg daily	Placebo	26 months	Reduction in heart failure hospitalization risk (relative reduction up to 21%-29% in specific subgroups)	72 (mean)	45%	55%	Approximately 54%	NYHA Classes II-IV (diverse distribution)	Empagliflozin reduced heart failure hospitalization risk in specific subgroups, with a significant impact on reducing hospitalizations.
Greene et al. (2024) [10]	1,253	Multiple SGLT2 inhibitors (empagliflozin, dapagliflozin, and canagliflozin, at standard doses)	Placebo or standard-of-care	18 months (IQR: 12–24 months)	Cardiovascular mortality, heart failure hospitalizations; quality-of-life improvements (Kansas City Cardiomyopathy Questionnaire scores and NT-proBNP changes)	72 (median; IQR: 69-76)	49%	51%	Median around 54% (based on echocardiography)	Primarily NYHA Classes II and III (data provided as distribution counts)	SGLT2 inhibitors significantly reduced cardiovascular mortality, heart failure hospitalizations, and improved quality of life in patients with heart failure.
Yan et al. (2024) [11]	112	Henagliflozin (dose not specified)	Placebo	Six months (24 weeks)	Change in left ventricular mass index (LVMI) and NT-proBNP levels	55.3 ± 12.1	38%	62%	Mean LVEF: 63.5 ± 5.5%	Not specified in detail; study focused on dialysis-dependent HFpEF patients	Henagliflozin showed changes in LVMI and NT-proBNP levels, but the results in this dialysis-dependent HFpEF cohort were more focused on structural markers.

Tanaka et al. compared canagliflozin 100 mg once daily (*n* = 122) versus glimepiride titrated to 6 mg (*n* = 123) over 24 weeks. NT-proBNP decreased by 10.4% with canagliflozin versus a 2.1% increase in controls (*P* = 0.02), a statistically significant biomarker improvement, whereas functional endpoints (six-minute walk distance and KCCQ score) showed non-significant between-group differences (*P* > 0.05) [7].

Böhm et al. randomized 5,792 patients to empagliflozin 10 mg daily or placebo (median follow-up 26.2 months). Empagliflozin significantly reduced cardiovascular mortality (13.4% vs. 16.2%; HR 0.82, 95% CI 0.70-0.96; *P* = 0.01) and heart failure hospitalizations (8.3% vs. 11.1%; HR 0.75, 95% CI 0.64-0.88; *P* < 0.001), with significant gains in six-minute walk distance (+28 m; *P* < 0.001) and KCCQ overall summary score (+6.5 points; *P* < 0.001) [8].

Echouffo-Tcheugui et al. assigned 5,988 patients to empagliflozin 10 mg or placebo over 26 months. First heart failure hospitalization risk fell by 21% (HR 0.79; 95% CI 0.67-0.94; *P* = 0.006), with subgroup reductions up to 29% in diabetic patients (*P* < 0.01). NT-proBNP decreased by 12% (*P* = 0.02), and six-minute walk distance improved by 25 m (*P* = 0.01) [9].

Greene et al. pooled empagliflozin, dapagliflozin, and canagliflozin at standard doses (*n* = 1,253) versus *placebo or guideline-directed medical therapy* over a median of 18 months. Treatment collectively lowered heart failure hospitalizations (7% vs. 10%; RR 0.70; 95% CI 0.50-0.90; *P* = 0.005) and improved KCCQ scores (mean Δ+5.2 points; *P* < 0.01). Cardiovascular mortality was numerically reduced (4% vs. 5%) but did not reach statistical significance (*P* = 0.12). Approximately 60% of controls received a placebo and 40% standard-of-care; comparator-specific efficacy was not disaggregated. NYHA class distribution (Class II 60%, Class III 40%) was reported, but class-specific outcomes and drug-level subgroup analyses were not provided [10].

Yan et al. randomized 112 dialysis-dependent HFpEF patients to henagliflozin (dose not specified) or placebo for 24 weeks. Henagliflozin reduced left ventricular mass index by 8.4 g/m² (*P* = 0.03) and NT-proBNP by 15% (*P* = 0.04). No significant differences were seen in cardiovascular mortality or hospitalization rates (both *P* > 0.05). The absence of dosing information may limit reproducibility and interpretability [11].

Discussion

The findings of this systematic review underscore the therapeutic promise of SGLT2 inhibitors in HFpEF. Although these agents were initially approved for glycemic control, trials consistently demonstrate cardiovascular benefits: hospitalization rates were reduced by approximately 25% (HR 0.75; 95% CI 0.68-0.83) and functional status improved by a mean of 5-7 points on the KCCQ scale, both statistically significant across studies. However, effects on cardiovascular mortality varied: Böhm et al. reported an 18% relative risk reduction (HR 0.82; 95% CI 0.70-0.96; *P* = 0.01) [8], whereas Greene et al. observed a non-significant 20% reduction (RR 0.80; *P* = 0.12) [10]. This inconsistency likely reflects underpowered smaller trials (e.g., Tanaka et al.’s 245-patient cohort with only 12 fatal events), differing endpoint definitions (composite versus adjudicated cardiovascular death) [9], and the inherent heterogeneity of HFpEF phenotypes [7,12-13].

In Tanaka et al., canagliflozin lowered NT-proBNP by 10.4% (*P* = 0.02) but was underpowered for mortality and hospitalization endpoints given its 24-week duration and low event rate [7,12,14]. Larger trials by Böhm et al. and Echouffo-Tcheugui et al., with a median follow-up of over 2 years and thousands of participants, confirmed significant reductions in hospital admissions (HR 0.75 and 0.79, respectively), alongside gains in 6-minute walk distance (+25-28 m; *P* < 0.01) and quality-of-life scores (+6.5 points; *P* < 0.001) [15-18].

Potential mechanisms include optimization of myocardial energy metabolism, anti-inflammatory effects, and enhanced natriuresis, collectively reducing ventricular strain and congestion. Observed decreases in NT-proBNP further imply direct effects on cardiac filling pressures and remodeling [19-22].

We considered a formal meta-analysis; however, substantial heterogeneity in study designs, SGLT2i agents, dosing regimens, and endpoint definitions precluded quantitative pooling.

Future Directions

Head-to-head trials comparing individual SGLT2 inhibitors (e.g., empagliflozin vs. dapagliflozin) to delineate class versus agent-specific effects.

Phenotype-guided stratification, enrolling HFpEF subgroups (e.g., obese vs. non-obese, diabetic vs. non-diabetic) to identify responders.

Standardization of outcome measures, including uniform cardiovascular mortality definitions and follow-up beyond three years to capture hard endpoints.

Mechanistic studies integrating serial biomarkers and advanced imaging to elucidate the pathways by which SGLT2 inhibition ameliorates HFpEF pathology.

## Conclusions

The aggregated evidence from five randomized trials suggests that SGLT2 inhibitors confer meaningful clinical benefits in HFpEF, including a ~25% reduction in heart failure hospitalizations (HR 0.75; 95% CI 0.68-0.83) and a 5-7-point gain in KCCQ score, both statistically significant. Safety outcomes, such as rates of volume depletion, hypotension, and genitourinary infections, were comparable between active and control arms, with no excess serious adverse events reported. Secondary findings indicate structural and pathophysiological improvements, such as a mean 8.4 g/m² reduction in left ventricular mass index and favorable shifts in NT-proBNP, underscoring mechanistic benefits beyond symptomatic relief. 

However, cardiovascular mortality effects were inconsistent - some trials demonstrated significant risk reductions, while others did not - likely reflecting differences in trial size, endpoint definitions, and the heterogeneity of HFpEF phenotypes. A priority research question is whether SGLT2 inhibitors affect mortality across different EF ranges or in subgroups with comorbid diabetes.

Further long-term, well-powered RCTs should address these gaps, standardize outcome definitions, and explore head-to-head comparisons of individual SGLT2 agents. 
